# Mitochondrial Signatures in Circulating Extracellular Vesicles of Older Adults with Parkinson’s Disease: Results from the EXosomes in PArkiNson’s Disease (EXPAND) Study

**DOI:** 10.3390/jcm9020504

**Published:** 2020-02-12

**Authors:** Anna Picca, Flora Guerra, Riccardo Calvani, Federico Marini, Alessandra Biancolillo, Giovanni Landi, Raffaella Beli, Francesco Landi, Roberto Bernabei, Anna Rita Bentivoglio, Maria Rita Lo Monaco, Cecilia Bucci, Emanuele Marzetti

**Affiliations:** 1 Institute of Internal Medicine and Geriatrics, Università Cattolica del Sacro Cuore, 00168 Rome, Italyfrancesco.landi@unicatt.it (F.L.); roberto.bernabei@unicatt.it (R.B.); emanuele.marzetti@policlinicogemelli.it (E.M.); 2 Fondazione Policlinico Universitario “Agostino Gemelli” IRCCS, 00168 Rome, Italy; giovandi@libero.it (G.L.); annarita.bentivoglio@policlinicogemelli.it (A.R.B.); mariarita.lomonaco@policlinicogemelli.it (M.R.L.M.); 3 Department of Biological and Environmental Sciences and Technologies, Università del Salento, 73100 Lecce, Italy; guerraflora@gmail.com (F.G.); raffaella.beli@unisalento.it (R.B.); 4 Department of Chemistry, Sapienza Università di Roma, 00185 Rome, Italy; federico.marini@uniroma1.it; 5 Department of Physical and Chemical Sciences, Università degli Studi dell’Aquila, 67100 L’Aquila, Italy; alessandra.biancolillo@univaq.it; 6 Institute of Neurology, Università Cattolica del Sacro Cuore, 00168 Rome, Italy

**Keywords:** aging, biomarkers, mitophagy, mitochondrial dynamics, mitochondrial quality control, mitochondrial-derived vesicles, exosomes, mitochondrial-lysosomal axis

## Abstract

Systemic inflammation and mitochondrial dysfunction are involved in neurodegeneration in Parkinson’s disease (PD). Extracellular vesicle (EV) trafficking may link inflammation and mitochondrial dysfunction. In the present study, circulating small EVs (sEVs) from 16 older adults with PD and 12 non-PD controls were purified and characterized. A panel of serum inflammatory biomolecules was measured by multiplex immunoassay. Protein levels of three tetraspanins (CD9, CD63, and CD81) and selected mitochondrial markers (adenosine triphosphate 5A (ATP5A), mitochondrial cytochrome C oxidase subunit I (MTCOI), nicotinamide adenine dinucleotide reduced form (NADH):ubiquinone oxidoreductase subunit B8 (NDUFB8), NADH:ubiquinone oxidoreductase subunit S3 (NDUFS3), succinate dehydrogenase complex iron sulfur subunit B (SDHB), and ubiquinol-cytochrome C reductase core protein 2 (UQCRC2)) were quantified in purified sEVs by immunoblotting. Relative to controls, PD participants showed a greater amount of circulating sEVs. Levels of CD9 and CD63 were lower in the sEV fraction of PD participants, whereas those of CD81 were similar between groups. Lower levels of ATP5A, NDUFS3, and SDHB were detected in sEVs from PD participants. No signal was retrieved for UQCRC2, MTCOI, or NDUFB8 in either participant group. To identify a molecular signature in circulating sEVs in relationship to systemic inflammation, a low level-fused (multi-platform) partial least squares discriminant analysis was applied. The model correctly classified 94.2% ± 6.1% PD participants and 66.7% ± 5.4% controls, and identified seven biomolecules as relevant (CD9, NDUFS3, C-reactive protein, fibroblast growth factor 21, interleukin 9, macrophage inflammatory protein 1β, and tumor necrosis factor alpha). In conclusion, a mitochondrial signature was identified in circulating sEVs from older adults with PD, in association with a specific inflammatory profile. In-depth characterization of sEV trafficking may allow identifying new biomarkers for PD and possible targets for personalized interventions.

## 1. Introduction

Parkinson’s disease (PD) is the second most common neurodegenerative disease affecting older adults [1]. Among neurodegenerative disorders, PD has shown the fastest growth in prevalence, due to global population aging, greater exposure to environmental risk factors, and longer disease duration [2,3].

Progressive demise of midbrain dopaminergic neurons of the *substantia nigra pars compacta* and dopamine depletion in the *striatum* are pathologic hallmarks of PD, which is characterized clinically by motor (i.e., bradykinesia, postural inability, rigidity, and tremor) and non-motor signs and symptoms (e.g., constipation, depression, sleep disorders, cognitive dysfunction) [4]. Dopaminergic neurotoxicity triggered by aggregation of misfolded α-synuclein is a well-established pathologic trait of PD [5]. However, the molecular events underlying the onset and progression of PD are still debated [5].

Mitochondrial dysfunction is a major factor in the pathogenesis of familial PD [6]. Age-related mitochondrial dyshomeostasis and the ensuing oxidative stress also favor aberrant protein folding and accrual of noxious protein aggregates, including α-synuclein [7]. The co-occurrence of mitochondrial dysfunction and impaired proteostasis during aging is therefore proposed as a mechanism triggering neuronal dysfunction in PD [8]. Remarkably, peripheral changes (e.g., systemic inflammation, metabolic alterations) are thought to precede and contribute to neurodegeneration in PD [9,10]. However, whether and how mitochondrial dysfunction and protein dyshomeostasis in neurons are linked to peripheral processes is currently unknown.

Failing mitochondrial quality control (MQC) processes is acknowledged as a major mechanism underlying mitochondrial dysfunction and loss of mitochondrial DNA (mtDNA) stability during aging and in the setting of neurodegeneration [11,12]. Extracellular vesicles (EVs) are delivery systems through which cells communicate or remove unwanted materials. Among EVs, exosomes originate from endocytic compartments [13]. Exosome precursors, referred to as intraluminal vesicles, are generated from the inward budding of small domains of early endosomal membranes. The accumulation of intraluminal vesicles into endocytic organelles results in the formation of multivesicular bodies (MVBs). MVBs release their cargo—now defined as exosomes—into the extracellular space via fusion with the plasma membrane [13,14,15]. Here, EV cargo may trigger inflammation [16]. In the setting of failing mitochondrial fidelity pathways, the generation and release of mitochondrial-derived vesicles (MDVs) may act as a further process of MQC orchestrated by mitochondrial–lysosomal crosstalk [17]. Although the release of MDV clears out dysfunctional organelle and avoids the permanence of noxious material within the cell, it may trigger a sterile inflammatory response by binding and activating membrane or cytoplasmic pattern recognition receptors (PRRs) (reviewed in [18]). Indeed, extracellular mtDNA can ignite an inflammatory response through the binding of hypomethylated CpG motifs, similar to those of bacterial DNA, to PRRs. This event could represent a mechanism linking mitochondrial dysfunction to systemic inflammation in PD [19,20,21]. However, mtDNA might not be the only mitochondrial component displaced into the systemic circulation via EVs to fuel systemic inflammation.

To shed light on the relationship between EV trafficking and inflammation in PD, we purified and characterized the cargo of small EVs (sEVs)/exosomes from the serum of older adults with PD and measured the concentration of a panel of circulating inflammatory biomarkers. Low level-fused (multi-platform) partial least squares discriminant analysis (PLS-DA) was applied to identify the molecular signature related to circulating sEVs and systemic inflammation in PD.

## 2. Materials and Methods

### 2.1. Study Design and Participants

The EXsomes in PArkiNson Disease (EXPAND) study was designed as a case-control investigation aimed at characterizing the cargo of circulating sEVs/exosomes in older adults with PD [21]. The protocol was approved by the Ethics Committee of the Università Cattolica del Sacro Cuore (Rome, Italy) (protocol # 0045298/17). The study was conducted in agreement with legal requirements and international norms (Declaration of Helsinki, 1964).

Participant recruitment was coordinated by the Institute of Neurology at the Università Cattolica del Sacro Cuore, (Rome, Italy) and was carried out at the Fondazione Policlinico Universitario “Agostino Gemelli” IRCCS (Rome, Italy). Analyses were conducted in a convenience sample of 28 participants, 16 cases diagnosed with PD according to the Queen Square Brain Bank criteria [22] under stable dopaminergic therapy for at least 1 month prior to enrolment, and 12 age- and sex-matched controls without any signs of parkinsonism or potential premotor symptoms. As previously detailed [10], drug-induced parkinsonism (dopamine receptor blocker or dopamine-depleting agent) or vascular (arteriosclerotic) parkinsonism, progressive neurological diseases, and cognitive impairment (i.e., Mini Mental State Examination (MMSE) score < 24/30) were considered exclusion criteria for both cases and controls. Prior to enrolment, all participants signed an informed consent form.

### 2.2. Blood Sampling and Serum Separation

Blood samples were collected in the morning by venipuncture of the median cubital vein after overnight fasting, using commercial collection tubes (BD Vacutainer; Becton, Dickinson and Co., Franklin Lakes, NJ, USA). Serum separation was obtained after 30 min of clotting at room temperature and subsequent centrifugation at 1000× *g* for 15 min at 4 °C. The upper clear fraction (serum) was collected in 0.5-mL aliquots. One aliquot was immediately delivered to the centralized diagnostic laboratory of the Fondazione Policlinico Universitario “Agostino Gemelli” IRCCS for standard blood biochemistry. The remaining aliquots were stored at −80 °C until analysis.

### 2.3. Isolation and Characterization of Small Extracellular Vesicles/Exosomes

#### 2.3.1. Purification of Small Extracellular Vesicles/Exosomes

Purification of sEVs/exosomes was performed as previously described [21]. Briefly, serum samples were diluted with equal volumes of phosphate-buffered saline (PBS) to reduce fluid viscosity and centrifuged at 2000× *g* at 4 °C for 30 min. Pellets were discarded to remove cell debris, and supernatants were collected and centrifuged at 12,000× *g* at 4 °C for 45 min to remove apoptotic bodies, mitochondrial fragments, cell debris, and large vesicles (mean size > 200 nm). After discarding pellets, supernatants were ultracentrifuged at 110,000× *g* at 4 °C for 2 h. Afterwards, pellets were recovered and resuspended in PBS, filtered through a 0.22-μm filter, and further ultracentrifuged at 110,000× *g* at 4 °C for 70 min to eliminate contaminant proteins. Pellets enriched in purified sEVs/exosomes were resuspended in 100 μL of PBS and proteins were quantified by the Bradford assay [23]. The amount of sEVs was normalized for total serum protein concentration and is shown as percentage of the control group set at 100%. For quality control purposes, sEVs/exosomes from one control and one PD participant were purified through a precipitation method using the miRCURY Exosome Serum/Plasma Kit (Qiagen, Hilden, Germany).

#### 2.3.2. Western Immunoblot Analysis of Small Extracellular Vesicles

The identification of sEV type and the characterization of protein cargo were accomplished by Western immunoblotting, as described elsewhere [24]. Briefly, equal amounts (1.25 μg) of sEV proteins from PD patients and controls were separated by sodium dodecyl sulphate polyacrylamide gel electrophoresis (SDS-PAGE) and subsequently electroblotted onto polyvinylidenefluoride (PVDF) Immobilon-P membranes (Millipore, Burlington, MA, USA). To determine the type of sEVs, membranes were probed with primary antibodies against CD9, CD63, and CD81 according to the criteria proposed by Kowal et al. [25]. As recommended by the International Society of Extracellular Vesicles [26], the purity of the sEV preparations obtained by ultracentrifugation or precipitation was also ascertained by probing samples for the cytosolic protein flotilin (positive control) and for heterogeneous nuclear ribonucleoprotein A1 (HNRNPA1, negative control).

Small EV protein cargo was characterized using antibodies targeting components of the five complexes of the mitochondrial electron transport chain [adenosine triphosphate 5A (ATP5A; complex V), mitochondrial cytochrome C oxidase subunit I (MTCOI; complex IV), nicotinamide adenine dinucleotide reduced form (NADH):ubiquinone oxidoreductase subunit B8 (NDUFB8; complex I), NADH:ubiquinone oxidoreductase subunit S3 (NDUFS3; complex I), succinate dehydrogenase complex iron sulfur subunit B (SDHB; complex II), and ubiquinol-cytochrome C reductase core protein 2 (UQCRC2; complex III)]. Technical specifications of the primary antibodies used are listed in Table 1. Membranes were incubated overnight and then probed for 1 h at room temperature with anti-mouse peroxidase-conjugated secondary antibodies (1:2000) (Bio-Rad Laboratories, Inc., Hercules, CA, USA). Blots were visualized using the ECL Plus Western blot substrate (Bio-Rad Laboratories) and ECL films (GE Healthcare, Chicago, IL, USA). Images were then acquired with an Epson Perfection V600 Scanner (Epson, Suwa, Japan) and bands were quantified by densitometry using the ImageJ software version 1.5Oi (National Institute of Health, Bethesda, MD, USA).

Values of optical density (OD) of immunodetected protein bands were normalized for the amount of sEV total proteins, as determined by the Bradford assay, and related to the control group, whose OD was set at 100%.

### 2.4. Measurement of Serum Concentrations of Inflammatory and Neurotrophic Biomolecules

A biomarker panel was designed on the basis of previous studies by our group in older adult populations [27,28]. Serum samples from PD and control participants were assayed in duplicate for a panel of 27 inflammatory mediators, including cytokines, chemokines, and growth factors using the Bio-Plex Pro Human Cytokine 27-plex Assay kit (#M500KCAF0Y, Bio-Rad Laboratories) on a Bio-Plex System with Luminex xMap Technology (Bio-Rad Laboratories) (Table 2). Data were acquired on Bio-Plex Manager Software 6.1 (Bio-Rad Laboratories) with instrument default settings. Standard curves across all analytes were optimized, outliers were removed, and results were recorded as concentration (pg/mL).

Serum levels of C-reactive protein (CRP), myeloperoxidase (MPO), fibroblast growth factor 21 (FGF21), and brain-derived neurotrophic factor (BDNF) were assayed by commercially available kits on an ELLA automated immunoassay system (Bio-Techne, San Jose, CA, USA) according to the manufacturer’s instructions.

### 2.5. Statistical Analysis

Descriptive statistics were run on all data. Differences in demographic, anthropometric, and clinical parameters between PD and control participants were assessed via *t*-test statistics and χ^2^ or Fisher’s exact tests, for continuous and categorical variables, respectively. All tests were two-sided, with statistical significance set at *p* < 0.05. Descriptive analyses were performed using the GraphPrism 5.03 software (GraphPad Software, Inc., San Diego, CA, USA).

To determine the circulating biomolecule profile of PD and control participants, multivariate analysis was performed through PLS-DA and soft independent modeling of class analogies (SIMCA). Multivariate statistics were conducted using functions written in-house and run under Matlab environment (release R2015b, The Mathworks, Natick, MA, USA).

#### 2.5.1. Partial Least Squares Discriminant Analysis

To explore whether it could be possible to classify the PD condition and identify a molecular signature in circulating EVs related to systemic inflammation, a multivariate analytical strategy was enacted [29]. This strategy was based on coupling a classification method (PLS-DA) with extensive validation of both the model performance and the identified biomarkers. PLS-DA operates by building a regression model between the predictors **X** (measured variables) and a dummy binary vector **y** coding for class belonging (in the present study, PD and controls). Regression was carried out through the PLS algorithm, which was based on projecting variables onto a low-dimensional subspace of latent variables (LVs) characterized by being the directions of maximum covariance between the **X** and the **y**, so as to overcome the problems inherent in dealing with a relatively high number of high correlated predictors. Classification was then accomplished by setting a threshold value on the predicted response, so that if the predicted **y** was higher than the threshold, the individual was classified as PD, or otherwise he/she was recognized as a control.

In order to properly validate the results of the classification strategy and to rule out the possibility of chance correlations, the PLS-DA model was validated by repeated double cross-validation (rDCV) and permutation tests [30,31]. In rDCV, two nested cross-validation loops are used to obtain the outcomes of external validation of the model performance (which is accounted for by the external loop) as independent as possible from the model selection stages (which are based on the results of the inner loop). The procedure is repeated a certain number of times (30 in the present study) to avoid the fact that the outcomes may depend on a single data split. Furthermore, to exclude the fact that good results could be due to chance, the values of the three figures of merit (number of misclassifications (NMC), area under the receiver operating characteristic curve (AUROC), and discriminant Q2 (DQ2)), which summarize the double-cross validated classification performances, were compared with their respective distributions under the null hypothesis (which were estimated by a permutation test with 1000 randomizations) [32].

Once the PLS-DA model was built and its predictive ability was tested and validated, model parameters could be inspected to identify potential discriminant biomarkers. Among the possible tools for model interpretation and identification of candidate biomarkers, variable importance in projection (VIP) [33] and rank product (RP) [31] indices were chosen for the present study. VIP scores account for the covariance between the predictors and the response by “apportioning” the variance in the response accounted for by the PLS-DA model to the individual experimental variables. VIP scores were scaled so that a “greater than 1” rule could be used to assess statistical significance. RP, instead, resulted from a model-based ranking of the predictors and accounted for how consistently a variable emerged as relevant in the resampling procedure. RP calculation relied on estimating the discriminant ability of predictors by means of the absolute value of the corresponding PLS-DA regression coefficient. The name RP derives from the fact that, at each iteration of the resampling procedure (in our case, of the rDCV), the absolute values of the PLS-DA regression coefficients are used to rank variables in decreasing order of discriminant ability. The predictor associated with the regression coefficient with the highest absolute value (greatest discriminant power) is given rank 1, the next larger 2, and so on. For each variable, RP is defined as the geometric mean of its ranks in all resampling (rDCV) segments. Predictors with the lowest RPs are identified as potential biomarkers. A more detailed description of PLS-DA and rDCV procedures may be found elsewhere [34].

#### 2.5.2. Soft Independent Modeling of Class Analogies

Soft independent modeling of class analogies (SIMCA) falls within the domain of chemometric class modeling techniques, that is, techniques that investigate a single category at a time and test how likely it is for an individual to be part of a specific class or not [35,36]. The model of each category is built by principal component analysis on the class only data, so that, in order to evaluate whether an individual may be considered as coming from that class or not (i.e., be accepted by the class model or not), a distance to the model is defined as

(1) $$d_{ic}=\sqrt{{\left(T_{ic,red}^{2}\right)}^{2}+{\left(Q_{ic,red}\right)}^{2}},$$

where $T_{ic,red}^{2}$ is the Mahalanobis distance of the *i*th sample from the center of the principal component (PC) space calculated for class *c*, $Q_{ic,red}$ is the orthogonal distance (residual) of the sample from its projection on the PC space of class *c*, and the subscript red indicates that the two statistics are normalized by their respective 95th percentile in order to be made comparable. Accordingly, classification of the unknown samples is achieved by setting a threshold (usually equal to $\sqrt{2}$) to the distance described in Equation (1): if $d_{ic}<\sqrt{2}$, then the individual is accepted by the class model; otherwise he/she is rejected.

## 3. Results

### 3.1. Characteristics of the Study Participants

A total of 28 participants were included in the study—16 older adults with PD and 12 age- and sex-matched controls. Demographic, anthropometric, and clinical characteristics of study participants are presented in Table 3. Age, sex distribution, MMSE score, and number of co-morbid conditions and medications did not differ between groups. Participants with PD had lower body mass index than controls, whereas serum albumin and total serum protein concentrations were comparable between groups.

### 3.2. Characterization of Small Extracellular Vesicles in Serum of Participants with and without Parkinson’s Disease

#### 3.2.1. Characterization of Small Extracellular Vesicles

**Figure 1:**
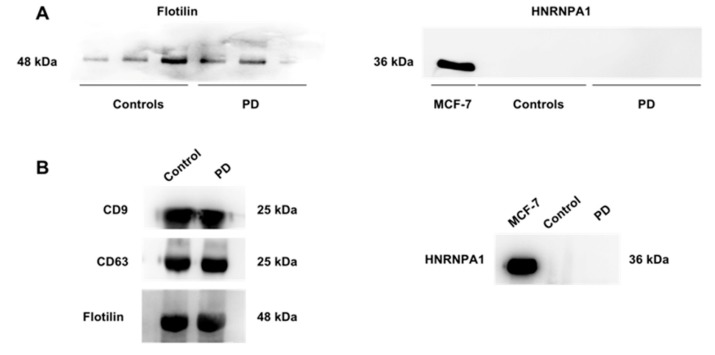
Representative blots of preliminary characterization of small extracellular vesicles (sEVs). (**A**) Blots of the cytosolic protein flotilin and ribonucleoprotein (HNRNPA1) as positive and negative markers, respectively, in purified sEVs obtained by ultracentrifugation from controls and participants with Parkinson’s disease (PD). MCF-7 cell extract was used as the positive control for the anti-HNRNPA1 antibody. (**B**) Blots of tetraspanins CD9 and CD63, flotilin, and HNRNPA1 in purified sEVs obtained from one control and one PD participant using a commercial precipitation kit.

The sEV nature of serum preparations obtained by ultracentrifugation or precipitation was ascertained by verifying the presence of three transmembrane proteins (i.e., CD9, CD63, and CD81) and one cytosolic protein (flotilin), and the absence of non-sEV components (i.e., HNRNPA1) [25,26]. As shown in Figure 1, both isolation methods yielded purified sEVs.

#### 3.2.2. Quantification of the Amount of Circulating Small Extracellular Vesicles

**Figure 2:**
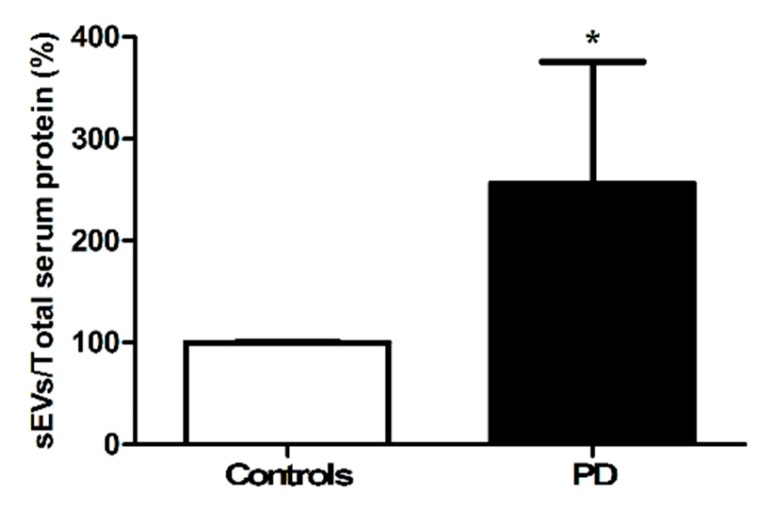
Levels of purified small extracellular vesicles (sEVs) in serum of controls (*n* = 12) and participants with Parkinson’s disease (PD; *n* = 16). Data were normalized for the amount of total serum protein and are shown as percentage of the control group set at 100%. Bars represent mean values (± standard deviation of the mean). * *p* < 0.0001 vs. controls.

The total amount of sEVs was significantly greater in PD participants relative to controls (*p* < 0.0001, Figure 2).

**Figure 3:**
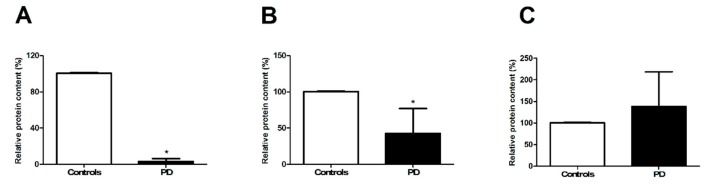
Protein expression of (**A**) CD9, (**B**) CD63, and (**C**) CD81 in purified small extracellular vesicles (sEVs) from controls (*n* = 12) and participants with Parkinson’s disease (PD; *n* = 16). Data were normalized for the amount of sEV total proteins and are shown as percentage of the control group set at 100%. Bars represent mean values (± standard deviation of the mean). Representative blots are shown in Figure S1. * *p* = 0.0001 vs. controls.

To characterize the population of sEVs in the two participant groups, protein expression levels of tetraspanins CD9, CD63, and CD81 were quantified in purified sEVs (Figure 3). Lower levels of CD9 and CD63 were found in participants with PD relative to controls (*p* < 0.0001, Figure 3A,B), whereas those of CD81 were unvaried between groups (*p* = 0.2215, Figure 3C).

#### 3.2.3. Characterization of the Cargo of Small Extracellular Vesicles

**Figure 4:**
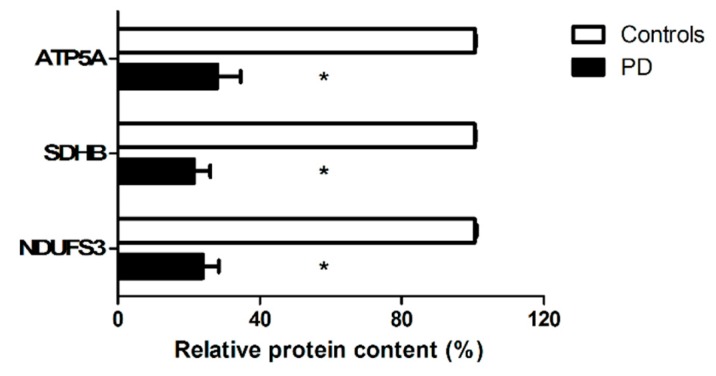
Protein expression of adenosine triphosphate 5A (ATP5A), succinate dehydrogenase complex iron sulfur subunit (SDHB), and nicotinamide adenine dinucleotide reduced form (NADH):ubiquinone oxidoreductase subunit S3 (NDUFS3) in purified small extracellular vesicles (sEVs) from controls (*n* = 12) and participants with Parkinson’s disease (PD; *n* = 16). Data were normalized for the amount of sEV total proteins and are shown as percentage of the control group set at 100%. Bars represent mean values (± standard deviation of the mean). Representative blots are shown in Figure S1. * *p* < 0.0001 vs. controls.

The protein cargo of sEVs was probed for the presence of selected mitochondrial markers [ATP5A (complex V), MTCOI (complex IV), NDUFB8 (complex I), NDUFS3 (complex I), SDHB (complex II), and UQCRC2 (complex III)]. Lower levels of ATP5A, NDUFS3, and SDHB were detected in sEVs from participants with PD compared with controls (Figure 4). No signal was retrieved for UQCRC2, MTCOI, or NDUFB8 in either participant group.

### 3.3. Identification of a Biomolecular Signature of Parkinson’s Disease by Partial Least Squares Discriminant Analysis

Serum levels of 37 biomolecules, including cytokines, chemokines, growth factors, tetraspanins, and mitochondrial markers, were analyzed through PLS-DA models built using a multi-matrix dataset on a low-level data fusion configuration. Prior to PLS-DA analysis, data from the different platforms were autoscaled, followed by normalization of each block by division by its Frobenius’ norm. Then, data from the various blocks were concatenated and a PLS-DA model was calculated and validated by rDCV as described in Section 2.5.1. Results are shown in Table 4.

The model correctly classified 94.2% ± 6.1% participants with PD and 66.7% ± 5.4% controls in the outer (external) cross-validation loop, corresponding to a classification ability of 82.4% ± 4.6% in the whole study population. The average AUROC was very close to 1. When compared with their distributions under the null hypothesis, all of the classification figures of merit were statistically significant (*p* < 0.0001).

Among the discriminant analytes identified by the PLS-DA model on the basis of inspection of VIP and RP scores, participants with PD showed lower levels of the sEV marker CD9, the mitochondrial subunit NDUFS3, the metabolic modulator FGF21, and the inflammatory cytokine interleukin 9 (IL9). In addition, participants with PD were characterized by higher serum concentrations of the inflammatory cytokines CRP and tumor necrosis factor alpha (TNF-α), and of the chemokine macrophage inflammatory protein (MIP) 1β (Table 4).

### 3.4. Verification of the Accuracy of Classification by Soft Independent Modeling of Class Analogies

SIMCA was applied to circulating sEV data to obtain a better insight into the characteristics of the PD condition. As described in Section 2.5.2., SIMCA builds and validates individual models for each category of interest and, therefore, allows for evaluation of how likely it is for a sample to belong to any of the modeled classes. Accordingly, SIMCA results are often expressed through two figures of merit (sensitivity and specificity)—the former indicates the percentage of samples from the model class correctly accepted by the model, whereas the latter refers to the percentage of individuals from other categories correctly rejected.

**Figure 5:**
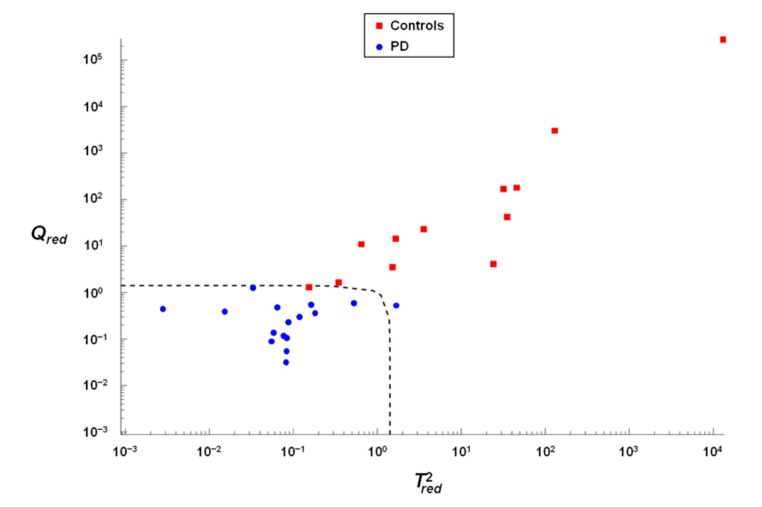
Soft independent modeling of class analogies modeling of Parkinson’s disease (PD) showing the projection of samples onto the spaces described by the statistical variables $T_{red}^{2}$ and $Q_{red}$. The dashed line indicates the threshold for acceptance $d=\sqrt{2}$.

A SIMCA model was built for the PD category and the optimal number of PCs was selected as the one offering the highest efficiency (geometrical average of sensitivity and specificity) in cross-validation. The model is graphically displayed in Figure 5, showing the projection of samples onto the model space described by the variables $T_{red}^{2}$ and $Q_{red}$ [see also Equation (1)]. A 93.8% sensitivity and 91.7% specificity in calibration, and 87.5% sensitivity and 93.8% specificity in cross-validation were determined.

## 4. Discussion

Peripheral processes (e.g., inflammation) and neuronal mitochondrial dysfunction contribute to neurodegeneration in PD [37,38,39,40]. However, the molecular determinants linking the two processes are underexplored. To help fill this gap in knowledge, we characterized the type and protein cargo of sEVs purified from the serum of elderly people with and without PD.

A greater amount of sEVs (Figure 2) and lower protein content of the two tetraspanins CD9 and CD63 were found in sEVs from participants with PD compared with controls (Figure 3). The protein cargo of sEVs in PD participants was characterized by lower levels of the mitochondrial components ATP5A (complex V), NDUFS3 (complex I), and SDHB (complex II) (Figure 4). The assessment of total protein content of purified sEVs enabled the determination of the overall quantity of the mixed sEV population (Figure 2). On the other hand, the presence of the three tetraspanins CD9, CD63, and CD81 in the purified sEV fraction allowed these vesicles to be identified as endosome-derived exosomes originating from the fusion of MVBs with the plasma membrane [25]. Notably, the identification of mitochondrial signatures indicated the presence of MDVs among sEVs.

MDVs are generated through the selective incorporation of protein cargoes, including outer and inner membranes and matrix content, and may serve as an additional MQC pathway [41]. Indeed, the generation and release of MDVs orchestrated by mitochondrial–lysosomal crosstalk may be triggered as a mechanism to clear out dysfunctional organelles and avoid permanence of noxious material within the cell [17]. Hence, the increased sEV secretion in PD (Figure 2) might have reflected the cell’s attempt to dispose dysfunctional mitochondria. In this scenario, the lower secretion of MDVs detected in PD participants (Figure 4) may indicate that the MQC flux was impaired in PD. This finding is in keeping with previous reports showing an association between PD and altered expression of genes encoding proteins involved in mitochondrial homeostasis via quality control mechanisms [e.g., Parkin, phosphatase and tensin homolog (PTEN)-induced putative kinase 1 (PINK1), DJ-1, leucine-rich repeat kinase 2 (LRRK2), ATPase 13A2, vacuolar protein sorting-associated protein 35 (VPS35)) [37,42]. Damaged MDV cargoes may also be delivered to lysosomes for degradation [41]. In support to this hypothesis, alterations of lysosomal function concomitant with impaired mitochondrial biogenesis have been described in Parkin gene (*PARK2*) mutated fibroblasts from a young patient with PD [38]. These changes were likely sustained by a mitochondrial genetic defect, blocking mitochondrial turnover and triggering premature cellular senescence [38].

Relevant insights into the association of mitochondrial dysfunction with peripheral changes in PD were obtained by integrating mitochondrial and inflammatory markers in the multi-platform PLS-DA analysis, which allowed for the accurate distinguishing of people with PD from controls. Seven biomolecules (i.e., CD9, NDUFS3, CRP, FGF21, IL9, MIP-1β, and TNF-α) were identified as relevant for the discrimination process (Table 4). NDUFS3 is a nuclear-encoded component of mitochondrial complex I, mutations of which are associated with defective complex I activity [43,44]. A large array of clinical conditions, ranging from lethal neonatal diseases to adult-onset neurodegenerative disorders including some forms of PD, show impaired oxidative phosphorylation primarily as a consequence of complex I [45] and III deficiency [46]. In the setting of PD, dysfunction of these mitochondrial subunits together with their insufficient removal via decreased MDV secretion may contribute to protein misfolding via increased oxidative stress [47].

The presence of FGF21 among the discriminant biomolecules in our model is especially remarkable. FGF21, besides being involved in a plethora of metabolic processes [48], has recently been related to dysfunctional MQC in neurons [49]. When mitochondrial dynamics become impaired, neurons activate a multibranched stress response that culminates in the release of FGF21 [49]. The induction of neuron-derived FGF21 has also been detected in brains of mouse models of tauopathy and prion disease [49]. Hence, FGF21 has been attributed a role as mitokine and has been proposed as a candidate marker of brain mitochondrial dysfunction [49].

The identification of a pattern of systemic inflammatory markers in participants with PD (i.e., lower levels of IL9 and higher concentrations of CRP, MIP-1β, and TNF-α) suggests the existence of an inflammatory signature of PD. This view is in keeping with previous reports indicating inflammatory perturbations in both sporadic and familial forms of PD [39,40]. Impairments of innate and adaptive immune response have been described in PD [50]. IL9 is a pleiotropic cytokine with pro-inflammatory and regulatory functions depending on the context in which it is induced and the nature of producing cells. IL9 influences the activity of different cell lines in both the immune and central nervous system (CNS). Notably, Th9 cells/IL9 signaling has been associated with neurodegeneration and autoimmune CNS diseases [51]. However, a neuroprotective role and support in repair functions have also been attributed to IL9 [51,52]. Accordingly, our finding of lower IL9 serum concentrations in PD individuals might suggest that dysregulated IL9 signaling could contribute to impaired neuroprotection/repair capacity in PD [53].

CRP is an acute phase protein commonly measured to monitor disease severity in acute and chronic inflammatory conditions. In advanced age, elevated circulating CRP concentrations independently predict morbidity, functional limitations, and mortality [54]. As such, CRP has been included within the panel of blood-based biomarkers to be implemented in geroscience-guided trials [55]. Noticeably, PD is associated with increased CRP levels in both peripheral blood and the cerebrospinal fluid [56]. Whether elevations in CRP contribute to neurodegeneration or occur as a result of an inflammatory response triggered by PD is presently unclear.

MIP-1β is involved in neurodegeneration by promoting CNS inflammation [57]. Remarkably, circulating MIP-1β levels have been associated with motor symptom severity, depression, and functional status, and have shown to predict their changes over time in a longitudinal study in older people with PD [58]. Finally, TNF-α is a key host defense and inflammatory cytokine that, under certain circumstances, can trigger cell death and tissue degeneration [59]. Our finding of higher serum levels of TNF-α in participants with PD is in line with the possibility that this biomolecule might be implicated in the pathophysiology of PD [60]. Indeed, TNF-α levels increase rapidly in experimental models of PD, and dopaminergic neurons are extremely sensitive to this cytokine [61]. Furthermore, specific polymorphisms in TNF gene characterized by higher TNF-α production are associated with earlier PD onset [62]. Remarkably, the incidence of PD in patients with inflammatory bowel disease was reduced by almost 80% in those exposed to anti-TNF therapy compared with patients who did not receive anti-TNF agents [63].

Albeit proposing novel findings, our study has limitations that need to be discussed. The investigation is associative in nature and cause–effect relationship between candidate mediators and PD pathophysiology cannot be established. Also, despite the fact that participants were carefully characterized, we cannot rule out the possibility that unknown comorbidities may have affected our results. Finally, although a fairly large number of analytes were assayed, it is possible that the inclusion of other biomolecules (e.g., α-synuclein) may provide additional insights into the relationships among sEV trafficking, inflammation, and mitochondrial dysfunction in PD.

Taken as a whole, findings from the present study support the hypothesis that alterations in MQC and release of MDVs may represent an unexplored mechanism through which mitochondrial dysfunction fuels systemic inflammation in PD [19,20,21]. In-depth characterization of exosomal trafficking may therefore allow identifying new biomarkers for PD and possible targets for interventions.

## Figures and Tables

**Table 1:** Technical specifications of the primary antibodies used for Western immunoblotting.

Antibody	Manufacturer and Catalog Number	Type	Species	Dilution	Detected Band MW (kDa)
ATP5A (complex V) MTCOI (complex IV) NDUFB8 (complex I) SDHB (complex II) UQCRC2 (complex III)	Abcam (Cambridge, MA, USA) ab1104413	Monoclonal	Mouse	1:250	55 40 20 30 48
CD9	Santa Cruz Biotechnology (Santa Cruz, CA, USA) (sc-13118)	Monoclonal	Mouse	1:200	25
CD63	Santa Cruz Biotechnology (sc-5275)	Monoclonal	Mouse	1:200	26
CD81	Santa Cruz Biotechnology (sc-166020)	Monoclonal	Mouse	1:200	25
NDUFS3 (complex I)	Santa Cruz Biotechnology (sc-374283)	Monoclonal	Mouse	1:200	25
Flotilin	Santa Cruz Biotechnology (sc-74566)	Monoclonal	Mouse	1:200	48
HNRNPA1	Santa Cruz Biotechnology (sc-32301)	Monoclonal	Mouse	1:1000	36

*Abbreviations*: ATP5A, adenosine triphosphate 5A; MTCOI, mitochondrial cytochrome C oxidase subunit I; HNRNPA1, heterogeneous nuclear ribonucleoprotein A1; MW, molecular weight; NDUFB8, nicotinamide adenine dinucleotide reduced form (NADH):ubiquinone oxidoreductase subunit B8; NDUFS3, NADH:ubiquinone oxidoreductase subunit S3; SDHB, succinate dehydrogenase complex iron sulfur subunit B; UQCRC2, ubiquinol-cytochrome C reductase core protein 2.

**Table 2:** Serum inflammatory biomediators assayed by multiplex immunoassay.

Biomarker Class	Assayed Biomolecules
**Cytokines**	IFNγ, IL1β, IL1Ra, IL2, IL4, IL5, IL6, IL7, IL8, IL9, IL10, IL12, IL13, IL15, IL17, TNF-α
**Chemokines**	CCL5, CCL11, IP-10, MCP-1, MIP-1α, MIP-1β
**Growth factors**	FGF-β, G-CSF, GM-CSF, PDGF-BB

Abbreviations: CCL, C-C motif chemokine ligand; FGF, fibroblast growth factor; G-CSF, granulocyte colony-stimulating factor; GM-CSF, granulocyte macrophage colony-stimulating factor; IFN, interferon; IL, interleukin; IL1Ra, interleukin 1 receptor agonist; IP: interferon-induced protein; MCP-1: monocyte chemoattractant protein 1; MIP: macrophage inflammatory protein; PDGF-BB, platelet-derived growth factor BB; TNF-α, tumor necrosis factor alpha.

**Table 3:** Main characteristics of study participants.

Characteristic	Controls (*n* = 12)	PD (*n* = 16)	*p* Value
Age (years), mean ± SD	75.5 ± 4.9	74.5 ± 8.4	0.6272
Gender (female), *n* (%)	5 (42)	9 (38)	0.4451
BMI (kg/m^2^), mean ± SD	29.2 ± 3.8	24.2 ± 3.0	0.010
Number of diseases *, mean ± SD	2.8 ± 2.1	3.2 ± 1.6	0.4621
Number of medications ^#^, mean ± SD	2.9 ± 2.0	3.4 ± 1.5	0.3729
MMSE score, mean ± SD	27.6 ± 2.4	27.4 ± 2.4	0.8171
Serum albumin (g/L), mean ± SD	41.6 ± 7.1	40.3 ± 3.9	0.5161
Total serum protein (g/L), mean ± SD	71.8 ± 4.6	72.9 ± 4.8	0.6541
Disease duration (months), mean ± SD	---	102.7 ± 69.1	
LEDD (mg), mean ± SD	---	587.6 ± 223.9	

Abbreviations: BMI: body mass index; LEDD: levodopa equivalent daily dose; MMSE: Mini Mental State Examination; PD: Parkinson’s disease; SD: standard deviation. * includes hypertension, coronary artery disease, prior stroke, peripheral vascular disease, diabetes, chronic obstructive pulmonary disease, and osteoarthritis. ^#^ includes prescription and over-the-counter drugs.

**Table 4:** Discriminant analytes identified by PLS-DA analysis.

	Controls (*n* = 12)	PD (*n* = 16)
CD9 (a.u.)	1133.4 (2710.4)	82.3 (53.2)
NDUFS3 (a.u.)	316.6 (881.8)	96.8 ± 128.0
CRP (mg/L)	0.5 (0.7)	1.5 (2.2)
FGF21 (pg/mL)	325.3 (392.0)	265.5 (151.8)
IL9 (pg/mL)	115.0 (27.1)	101.8 (3.6)
MIP-1β (pg/mL)	158.6 (97.5)	184.6 (23.5)
TNF-α (pg/mL)	31.2 (27.4)	42.2 (10.2)

Data are shown as median (interquartile range). Grey-shadowed rows correspond to extracellular vesicle-related marker and cargo; white rows correspond to inflammatory mediators. *Abbreviations*: a.u.: arbitrary unit; CRP: C-reactive protein; FGF21: fibroblast growth factor 21; IL9: interleukin 9; MIP-1β: macrophage inflammatory protein 1β; NDUFS3: nicotinamide adenine dinucleotide reduced form (NADH): ubiquinone oxidoreductase subunit S3; PD: Parkinson’s disease; PLS-DA: partial least squares discriminant analysis; TNF-α: tumor necrosis factor alpha.

## Supplementary Materials

The following is available online at https://www.mdpi.com/2077-0383/9/2/504/s1: Figure S1: Representative blots of biomolecules detected in purified small extracellular vesicles.
