# Supplementary material for: Mitochondrial Signatures in Circulating Extracellular Vesicles of Older Adults with Parkinson’s Disease: Results from the EXosomes in PArkiNson’s Disease (EXPAND) Study
Source: J Clin Med. 2020 Feb 12;9(2):504. doi: 10.3390/jcm9020504 (PMC7074517; doi:10.3390/jcm9020504)
Supplement: Supplementary file 1 [file jcm-09-00504-s001.pdf]

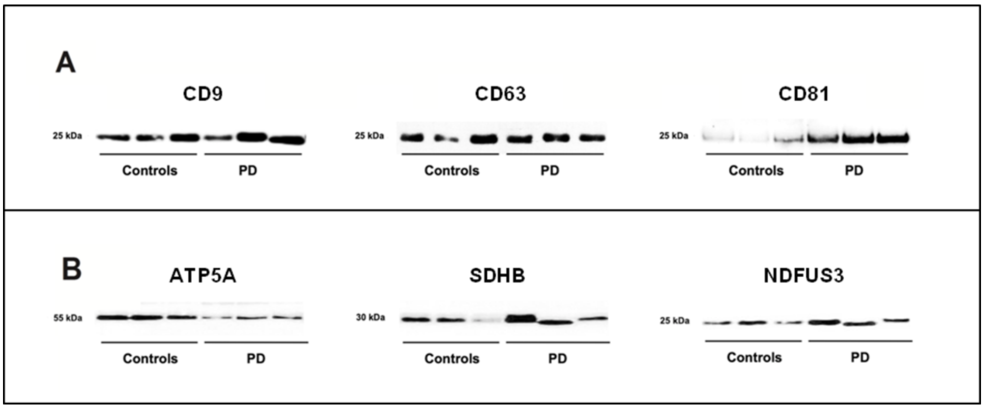

Figure S1. Representative blots of biomolecules detected in purified small extracellular vesicles. (**A**) Representative blots of tetraspanins CD9, CD63, and CD81 in purified small extracellular vesicles from controls and participants with Parkinson's disease (PD). (**B**) Representative blots of adenosine triphosphate 5A (ATP5A), succinate dehydrogenase complex iron sulfur subunit (SDHB), and nicotinamide adenine dinucleotide reduced form (NADH): ubiquinone oxidoreductase subunit S3 (NDUFS3) in purified small extracellular vesicles from controls and participants with PD.
For all biomolecules, optical density values were normalized for the amount of small extracellular vesicle total proteins, as determined by the Bradford assay.
